# Recombinant human luteinizing hormone co-treatment in ovarian stimulation for assisted reproductive technology in women of advanced reproductive age: a systematic review and meta-analysis of randomized controlled trials

**DOI:** 10.1186/s12958-021-00759-4

**Published:** 2021-06-21

**Authors:** Alessandro Conforti, Sandro C. Esteves, Peter Humaidan, Salvatore Longobardi, Thomas D’Hooghe, Raoul Orvieto, Alberto Vaiarelli, Danilo Cimadomo, Laura Rienzi, Filippo Maria Ubaldi, Fulvio Zullo, Carlo Alviggi

**Affiliations:** 1 University of Naples Federico II, Department of Neuroscience, Reproductive Science and Odontostomatology, University of Naples Federico II, Naples, Italy; 2 ANDROFERT, Andrology and Human Reproduction Clinic, Campinas, Brazil; 3 Department of Surgery, University of Campinas, Campinas, Brazil; 4 Faculty of Health, Aarhus University, Aarhus, Denmark; 5 Fertility Clinic, Skive Regional Hospital, Skive, Denmark; 6 Merck Serono S.p.A, Rome, Italy; 7 Department of Development and Regeneration, Biomedical Sciences Group, KU Leuven (University of Leuven), Merck, Leuven, Belgium; 8 KGaA, Darmstadt, Germany; 9 Department of Obstetrics and Gynecology, Chaim Sheba Medical Center, Ramat Gan, Israel; 10 The Tarnesby-Tarnowski Chair for Family Planning and Fertility Regulation, Sackler Faculty of Medicine, Tel-Aviv University, Tel-Aviv, Israel; 11 Clinica Valle Giulia, G.EN.E.R.A. Centers for Reproductive Medicine, Rome, Italy

**Keywords:** Luteinizing hormone, Recombinant luteinizing hormone, Assisted reproductive technology, In vitro fertilization, Advanced reproductive age

## Abstract

**Introduction:**

Several studies suggest that luteinizing hormone (LH) could improve IVF outcome in women of advanced reproductive age by optimizing androgen production. In this review, we assessed the role of recombinant-human LH (r-hLH) and recombinant human follicle stimulating hormone (r-hFSH) co-treatment in ovarian stimulation for assisted reproductive technology in women of advanced reproductive age candidates for assisted reproduction.

**Material and methods:**

Using a preregistered protocol we systematically searched Medline/PubMed, Scopus and the ISI Web of Science databases to identify randomized controlled trials in which r-hFSH monotherapy protocols were compared with r-hFSH/r-hLH co-treatment in women ≥35 years undergoing fresh IVF cycles. We calculated the pooled odds ratio (OR) for dichotomous data and the weight mean difference (WMD) for continuous data with an associated 95% confidence interval (CI). The meta-analyses were conducted using the random-effect model. *P* values < 0.05 were considered statistically significant. Subgroup analyses of all primary and secondary outcomes were performed only in women aged 35–40 years.

**Results:**

Twelve studies were identified. In women aged between 35 and 40 years, r-hFSH/r-hLH co-treatment was associated with higher clinical pregnancy rates (OR 1.45, CI 95% 1.05–2.00, I^2^ = 0%, *P* = 0.03) and implantation rates (OR 1.49, CI 95% 1.10–2.01, I^2^ = 13%, *P* = 0.01) versus r-hFSH monotherapy. Fewer oocytes were retrieved in r-hFSH/r-hLH-treated patients than in r-hFSH-treated patients both in women aged ≥35 years (WMD -0.82 CI 95% -1.40 to − 0.24, I^2^ = 88%, *P* = 0.005) and in those aged between 35 and 40 years (WMD -1.03, CI − 1.89 to − 0.17, I^2^ = 0%, *P* = 0.02). The number of metaphase II oocytes, miscarriage rates and live birth rates did not differ between the two groups of women overall or in subgroup analysis.

**Conclusion:**

Although more oocytes were retrieved in patients who underwent r-hFSH monotherapy, this meta-analysis suggests that r-hFSH/r-hLH co-treatment improves clinical pregnancy and implantation rates in women between 35 and 40 years of age undergoing ovarian stimulation for assisted reproduction technology. However, more RCTs using narrower age ranges in advanced age women are warranted to corroborate these findings.

**Supplementary Information:**

The online version contains supplementary material available at 10.1186/s12958-021-00759-4.

## Introduction

Female age is the most critical factor in fertility. Natural fecundity and pregnancy rate following assisted reproductive technology (ART) decrease dramatically in women aged 35 years and above [1, 2]. The age-related decrease in oocyte quality, which ultimately affects embryo quality and implantation, seems to be the driving factor of this phenomenon [3, 4]. Indeed, a retrospective analysis of 1296 embryos screened by next-generation sequencing indicated that the probability of blastocyst euploidy progressively decreases with every year of female age [3]. This effect is mainly due to the higher incidence of chromosomal abnormalities in oocytes of advanced-age women [5]. In addition, reproductive endocrine function progressively declines with advancing age [6]. In detail, this process seems to affect the luteinizing hormone (LH) system and androgen production [7–9]. The impact of ageing on fertility is highly relevant given that an increasing number of women seeking fertility treatment are of advanced reproductive age. Data from the European Society of Human Reproduction and Embryology (ESHRE) Registry (years 2002 to 2014) indicate that the percentage of fertility treatments in advanced age women (≥ 35 years of age) increased from 48.4 to 56.7%. In women ≥40 years, the increase was from 13.2 to 19.7% [10, 11]. Consistently, Ishihara et al. reported that the global proportion of women aged ≥40 years seeking ART treatment increased from 15.5 to 19.8% between 2006 and 2007 [12]. Moreover, in a study covering 2008, 2009 and 2010, the International Committee for Monitoring Assisted Reproductive Technologies reported that 57.1% of women (36.3% between 35 and 39 years old and 20.8% above 39 years) were treated with fresh cycles [13]. Delayed childbearing is widely practiced in both developed and developing nations, and is likely to increase [13–16]. Consequently, the focus now is to find measures to counteract the natural age-related decline in fertility [12, 14].

The use of elevated gonadotropin dosage is one of the most frequently used measures with which to increase reproductive outcomes in aged women [17]. However, this practice is not effective in women of advanced reproductive age with a low ovarian reserve [18]. r-hFSH/r-hLH co-treatment during ovarian stimulation (OS) could be more effective than r-hFSH alone in improving implantation and clinical pregnancy rates in women of advanced reproductive age [9, 19, 20]. Notably, r-hFSH/r-hLH co-treatment is used in the novel Duo-Stim stimulation protocol that includes both follicle and luteal phase stimulation [21–26]. The Duo-Stim approach significantly improved live birth rates in women fulfilling the Bologna criteria [26] and significantly contributed to the final transferable blastocyst yield in women with a low ovarian reserve [25].

Luteinizing hormone plays an important role in follicle growth by contributing to follicle maturation, fertilization and embryo quality [27]. It affects the endometrium by promoting the decidualization of endometrial stromal cells and embryo implantation [28]. Administered exogenously, LH promotes androgen production in theca cells [7, 29]. Consequently, the administration of r-hLH could increase androgenic and estrogenic follicle fluid levels, which are also often impaired in women of advanced reproductive age [29, 30]. On the other hand, it seems improbable that age-related chromosomal damage could be counteracted by LH action. The role of r-hFSH/r-hLH co-treatment in advanced age women is still debated [31, 32]. The mixed results observed among trials could reflect the fact that live births dramatically drop year by year in women above the age of 34 years [15, 33]. Indeed, women over 40 years generally have a lower probability of pregnancy leading to live birth than do women aged 35–39 years [11, 14, 33]. This finding has been largely attributed to the fact that embryo euploidy rates, which are the most important factor governing live birth following ART, are remarkably higher in women aged 35–39 years than in those above the age of 40 years [3, 33–35]. In other words, previous studies might have failed to find any effect of r-hLH because they did not include narrow age ranges [36, 37]. Therefore, we conducted a systematic review and aggregated the data of randomized controlled trials (RCTs) on the effect of r-hFSH/r-hLH co-treatment OS for ART in women of advanced reproductive age, focusing on a narrow age range of between 35 and 40 years.

## Material and methods

### Protocol and registration

This study was exempted from institutional review board approval as it did not involve any human intervention. We adhered to the Preferred Reporting Items for Systematic Reviews and Meta-Analyses (PRISMA) guidelines [38]. The study protocol was registered at http://www.crd.york.ac.uk/PROSPERO/ (registration number CRD 42020153814).

### Search strategy

We carried out a systematic search using Medline/PubMed, Scopus, and the ISI Web of Science to identify all relevant studies. The Boolean search criteria adopted included the MESH search terms *luteinizing hormone* OR ‘recombinant LH’ OR ‘r-hLH’ OR ‘r-hLH’ AND ‘ovulation induction’ OR *assisted reproductive technology* OR ‘ART’ OR ‘in vitro’ ‘fertilization’ OR ‘IVF’. Hand searches of review articles and reference lists were carried out. No time restriction or language restrictions were applied and the end date for all searches was June 2020. Case reports, conference proceedings, abstracts, doctoral theses, and dissertations were not considered.

### Eligibility and data extraction

We included only RCTs in which recombinant FSH (r-hFSH) alone protocols were compared to r-hFSH/r-hLH co-treatment in women aged 35 years or above undergoing fresh IVF cycles. Three authors (AC, DC, AV) conducted data extraction using predefined data fields.

### Outcome measures

The primary outcome was clinical pregnancy rate per started cycle defined according to the *International Glossary on Infertility and Fertility Care* [39] as “A pregnancy diagnosed by ultrasonographic visualization of one or more gestational sacs or definitive clinical signs of pregnancy”. Secondary outcomes were the number of oocytes retrieved, number of metaphase II (MII) oocytes, implantation rate (defined as the number of gestational sacs observed divided by the number of embryos transferred), live birth rate (defined as the number of deliveries per started cycles) and first trimester miscarriage rate. As safety outcome, we evaluated the risk of ovarian hyperstimulation syndrome (OHSS).

### Study selection

Study selection was conducted independently by three authors (AC, DC and AV). The titles and abstracts of all articles identified were scrutinized and the full texts of eligible articles were obtained. Disagreements regarding paper screening and eligibility were resolved by discussion.

### Bias assessment

Three authors (AC, DC and AV) independently assessed the risk of bias of the studies eligible for the review using the Cochrane risk of bias tool [40]. The following issues were assessed: 1) random sequence generation; 2) allocation concealment; 3) binding of participants and personnel; 4) incomplete outcome data; and 5) selective reporting. Differences in terms of baseline characteristics between groups were also assessed. For each issue, the risk of bias was graded as low, unclear or high. To assess the risk of bias across studies, we evaluated the funnel plots of primary outcomes both visually and formally using the trim-and-fill method and the Egger’s test [41, 42].

### Statistical analysis

Statistical analysis was carried out using Review Manager 5.4 (The Nordic Cochrane Centre, The Cochrane Collaboration). We calculated the pooled odds ratio (OR) for dichotomous data and weight mean difference (WMD) for continuous data with an associated 95% confidence interval (CI). Given that the studies included in this review differ significantly in terms of dosage used, protocols and ethnicity, we used a random effect model to account for sources of variations among studies because it is a more conservative approach than the fixed effect model [43]. Heterogeneity was assessed using the percentage of total variation in the estimated effect across studies (I^2^). *P* values < 0.05 were considered statistically significant.

### Subgroup and sensitivity analysis

Subgroup analyses of all primary and secondary outcomes were performed only in women aged between 35 and 40 years. Sensitivity analysis was carried out by measuring the overall effect size of all groups. Specifically, studies judged to be at a high risk of bias for at least one issue or to be at an unclear risk for at least two issues according to the risk of bias assessment tool were excluded from the analysis.

## Results

### Study selection and characteristics

**Fig. 1:**
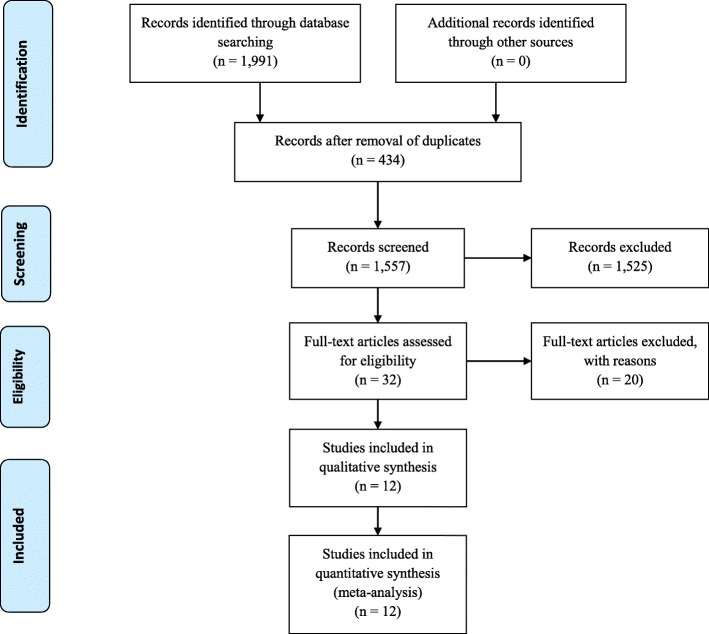
Study flow chart according to PRISMA guidelines

A total of 1991 papers were identified in the Medline/PubMed (*n* = 565), Scopus (*n* = 858) and Embase databases (*n* = 568) (Fig. 1). Duplications were removed by Endnote Online and manually (*n* = 434). Abstracts and titles (*N* = 1557) were reviewed by two authors (AC, CA). Disagreements were resolved by discussion with all authors. Thirty-two full-text papers were assessed for eligibility. Twenty studies were excluded because they did not fulfill the inclusion criteria [44–63]. Overall, 12 RCTs were identified [9, 20, 36, 37, 64–71]. The characteristics of the studies are shown in Table 1. Risk of bias within studies is illustrated in Supplemental Fig. 1.

**Table 1:** Characteristics of studies included

Reference	Study type	Period of observation	Country	Pituitary suppression	Population	Intervention	Comparison	Results
Humaidan et al. 2004 [20]	RCT subgroup analysis	November 2001 to October 2002	Denmark (single-centre)	GnRH agonist long Buserelin 0.5 mg /day	*N* = 39 Age 35–40 years	Group A1 *N* = 21 r-hFSH +r-hLH r-hLH started at stimulation day 8 r-hLH dosage r-hFSH r-hLH ratio 2:1	Group B1 *N* = 18 r-hFSH	Significantly higher implantation rate in r-hLH supplemented group
Marrs et al. 2004 [19, 69]	RCT subgroup analysis	n.a	USA (multi-centre)	GnRH agonist long Leuprolide 0.5 mg/day	*N* = 121 Age 35–40 years	Group A *N* = 65 r-hFSH +r-hLH r-hLH started at stimulation day 6 r-hLH dosage 150 IU	Group B *N* = 56 r-hFSH	Higher implantation rate and clinical pregnancy rate in r-hLH supplemented group
Fabregues et al. 2006 [67]	RCT	November 2003 to September 2004	Spain (single-centre)	GnRH agonist long Triptorelin 0.1 mg/day	*N* = 120 Age 35–42 years	Group A *N* = 60 r-hFSH + r-hLH r-hLH started at stimulation day 6 r-hLH dosage 150 IU	Group B *N* = 60 r-hFSH	Significantly lower number of oocytes retrieved and MII oocytes in r-hLH supplemented group Comparable implantation rate and pregnancy rates between groups
Barrenetxea et al. 2008 [70]	RCT	January to June 2005	Spain (single-centre)	GnRH agonist short Leuprolide 0.5 mg	*N* = 84 Age ≥ 40 years	Group A *N* = 42 r-hFSH + r-hLH r-hLH started at stimulation day 7 r-hLH dosage 150 IU	Group B *N* = 42 r-hFSH	Comparable implantation rate, pregnancy rates and number of oocytes retrieved between groups
N. Andersen et al. 2008 [65]	RCT subgroup analysis	August 2003 to November 2004	Denmark Sweden Norway Finland (multi-centre)	GnRH agonist long Nafarelin 200 μg twice a day	*N* = 100 Age 35–40 years	Group A *N* = 49 r-hFSH +r-hLH r-hLH started at stimulation day 6 r-hLH dosage 150 IU	Group B *N* = 51 r-hFSH	Comparable clinical pregnancy rate, implantation rate and number of oocytes retrieved between groups
Matorras et al. 2009 [64]	RCT	January 2005 to November 2006	Spain (single-centre)	GnRH agonist long Triptorelin 0.1 mg/day	*N* = 131 Age 35–39 years	Group B *N* = 63 r-hFSH + r-hLH r-hLH started at stimulation day 6 r-hLH dosage 150 IU	Group A *N* = 68 r-hFSH	Significantly higher implantation rate, live birth rate in r-hLH supplemented group. Higher clinical pregnancy rate in r-hLH supplemented group
Bosch et al. 2011 [9]	RCT	January 2005 to December 2007	Spain (single-centre)	GnRH antagonist Cetrorelix 0.25 mg/day OC during the cycle before stimulation	*N* = 292 Age 36–39 years	Group A *N* = 150 r-hFSH + r-hLH r-hLH dosage 75 IU r-hLH started at stimulation day 1	Group B *N* = 142 r-hFSH	Significantly lower number of oocytes retrieved in r-hLH supplemented group Significantly higher implantation rate in r-hLH supplemented group Higher clinical pregnancy rates in r-hLH supplemented group
Fabregues et al. 2011 [68]	RCT	January 2007 to February 2008	Spain (single-centre)	GnRH agonist long Triptorelin 0.1 mg /day	*N* = 187 Age 35–41 years	Group A1 *N* = 62 r-hFSH + r-hLH Group A2 *n* = 63 r-hFSH + r-hLH r-hLH started at stimulation day 6 in A1 and A2 Group A1 r-hLH dosage 37.5 IU Group A2 r-hLH dosage 75 IU	Group B *N* = 62 r-hFSH	Significantly lower number of oocytes retrieved and MII oocytes in r-hLH supplemented group Comparable implantation rate and pregnancy rates between groups
Konig et al. 2013 [36]	RCT	January 2004 to September 2010	Netherlands (multi-centre)	GnRH antagonist Cetrorelix 0.25 mg/day	*N* = 253 Age 35–43 years	Group A *N* = 125 r-hFSH + r-hLH r-hLH started at stimulation day 6 r-hLH dosage 150 IU	Group B *N* = 128 r-hFSH	Comparable clinical pregnancy rate, implantation rate and n oocytes retrieved between groups
Vuong et al. 2015 [37]	RCT	October 2012 to June 2014	Vietnam (single-centre)	GnRH antagonist Cetrorelix	*N* = 240 Age ≥ 35 years	Group A *N* = 120 r-hFSH + r-hLH r-hLH started at stimulation day 6 r-hLH dosage 75 IU	Group B *N* = 120 r-hFSH	Comparable clinical pregnancy rate, implantation rate and number of oocytes retrieved between groups
Younis et al. 2016 [66]	RCT	April 2010 to April 2013	Israel (single-centre)	GnRH antagonist Cetrorelix 0.25 mg/day	*N* = 62 Age 35–44 years	Group A *N* = 32 r-hFSH +r-hLH r-hLH started with antagonist r-hLH dosage r-hFSH r-hLH ratio 2:1	Group B *N* = 30 r-hFSH	Comparable clinical pregnancy rate, implantation rate and number of oocytes retrieved between groups
Humaidan et al. 2017 [71]	RCT	January 2014 and February 2015	Denmark (multi-centre)	GnRH agonist long Triptorelin acetate 0.1 mg	*N* = 821 Age 35–41 years BMI 18–31 kg/m2 Poor response according the Bologna criteria	Group A *N* = 405 r-hFSH + r-hLH r-hLH started at stimulation day 1 r-hLH dosage r-hFSH r-hLH ratio 2:1	Group B *N* = 416 r-hFSH	Comparable number of oocytes retrieved

Continuous data are presented as mean ± standard deviation or median value when specified; categorical data are presented as percentage or Odds Ratio

*n.a* not available, *r-hFSH* recombinant human follicle stimulating hormone, *r-hLH* recombinant human luteinizing hormone; ≈: comparable, *NS* no significant, *OR* odds ratio, *CI* confidence interval, *p p* value

### Synthesis of results

**Fig. 2:**
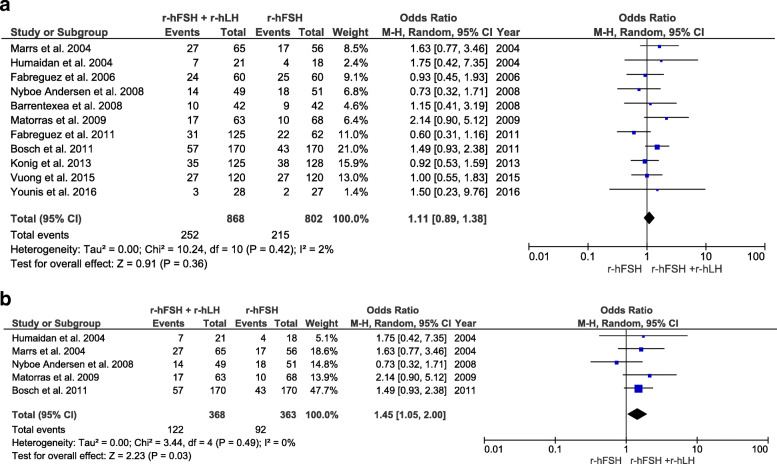
Forest plot showing the effect of r-hFSH + r-hLH versus r-hFSH monotherapy in ovarian stimulation on clinical pregnancy rates: **a** age ≥ 35 years, **b** between 35 and 40 years old

**Fig. 3:**
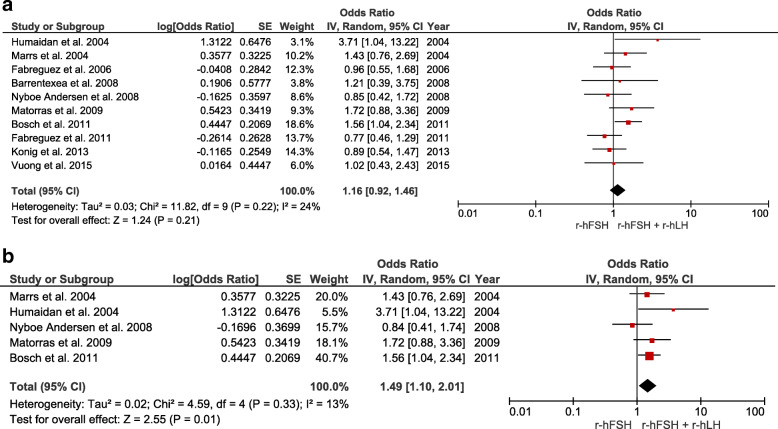
Forest plot showing the effect of r-hFSH + r-hLH versus r-hFSH monotherapy in ovarian stimulation on the implantation rate: **a** age ≥ 35 years, **b** between 35 and 40 years old

Clinical pregnancy rates were investigated in 11 RCTs (number of participants = 1670). Overall, no significant differences were observed between patients aged 35 years and above who underwent OS using r-hFSH alone protocols versus those who underwent r-hFSH/r-hLH co-treatment. By contrast, in the subgroup of women between 35 and 40 years of age, the clinical pregnancy rate was higher after r-hFSH/r-hLH co-treatment than after r-hFSH treatment alone (OR 1.45, CI 95% 1.05–2.00, I^2^ = 0%, *P* = 0.03) (Fig. 2). Implantation rates were investigated in 10 RCTs (number of participants = 1615). Overall, no significant differences were observed between groups. Conversely, the implantation rate was significantly higher in the subgroup analysis of women between the ages of 35–40 years who received r-hFSH/r-hLH co-treatment than in those receiving r-hFSH monotherapy (OR 1.49, CI 95% 1.10–2.01, I^2^ = 13%, *P* = 0.01) (Fig. 3).

**Fig. 4:**
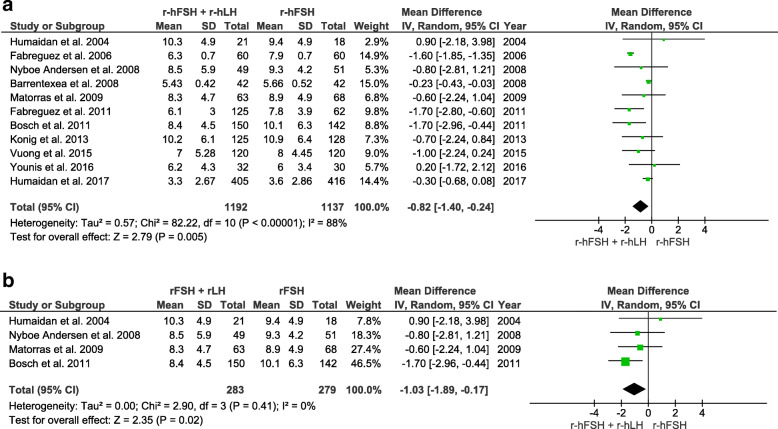
Forest plot showing the effect of r-hFSH + r-hLH versus r-hFSH monotherapy in ovarian stimulation on the total number of oocytes retrieved: **a** age ≥ 35 years, **b** between 35 and 40 years old

The number of oocytes retrieved was counted in 11 RCTs (number of participants = 2329). Overall, the number of oocytes retrieved was significantly lower in the r-hFSH/r-hLH co-treatment group than in the r-hFSH monotherapy group (WMD -0.82 CI 95% -1.40 to − 0.24, I^2^ = 88%, *P =* 0.005). Likewise, a significantly lower number of oocytes was retrieved in the r-hFSH/r-hLH group than in the r-hFSH alone group in a subgroup analysis of 35–40 year old women (WMD -1.03, CI − 1.89 to − 0.17, I^2^ = 0%, *P* = 0.02) (Fig. 4).

**Fig. 5:**
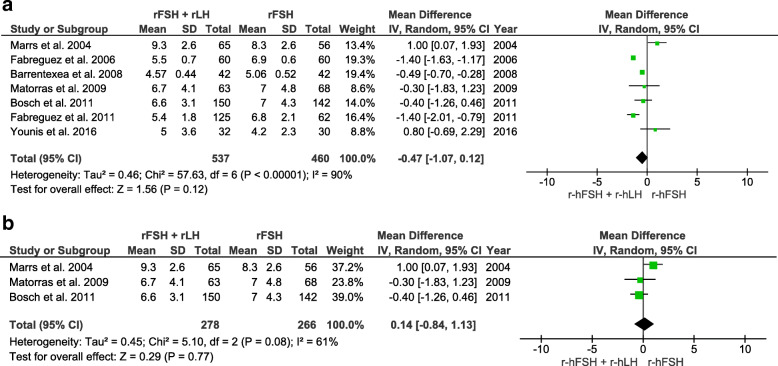
Forest plot showing the effect of r-hFSH + r-hLH versus r-hFSH monotherapy in ovarian stimulation on the number of metaphase II oocytes: **a** age ≥ 35 years, **b** between 35 and 40 years old

The number of MII oocytes was assessed in seven RCTs (number of participants = 997). No significant differences were observed in the overall analysis or in the subgroup analysis of 35–40 year old women (Fig. 5). Live birth rates were evaluated in only two RCTs (number of participants = 371), and no differences were detected between groups (Supplemental Fig. 2). Miscarriage rate was reported in seven RCTs (number of participants = 1023). The overall odds ratio did not reveal any differences between the r-hFSH plus r-hLH group and the r-hFSH alone group. Similar findings were observed in the subgroup analysis of 35–40 year old women (Supplemental Fig. 3). OHSS risk was reported in 4 RCTs (number of participants = 1420). The overall odds ratio did not reveal any significant difference between the r-hFSH/r-hLH group and r-hFSH alone group (Supplemental Fig. 4).

### Risk of bias across studies

The risk of a significant bias across studies regarding the primary outcome was excluded by Egger’s test (*p* = 0.69), and confirmed by visual inspection of the funnel plots and by the trim and fill method (Supplemental Fig. 5).

### Sensitivity analysis

Sensitivity analysis conducted excluding studies with a high risk of bias revealed that the pooled effect sizes were not affected by any outcome addressed (Supplementary Table 1).

## Discussion

### Summary of evidence

This meta-analysis suggests that r-hFSH/r-hLH co-treatment during OS increases both clinical pregnancy rates and implantation rates in women between the ages of 35 and 40 years who underwent OS for IVF. R-hFSH/r-hLH co-treatment was associated with fewer oocytes retrieved; however, no differences in terms of the number of MII oocytes retrieved were observed between groups. The miscarriage rate did not differ between the overall population and women aged 35–40 years. Unfortunately, only two RTCs reported live birth rate per started cycle [37, 64]. A benefit in terms of live births per cycle was reported in only one RCT in which only women between the ages of 35–39 years were enrolled [64].

### Interpretation of results and clinical considerations

Luteinizing hormone plays several pivotal roles during folliculogenesis. In fact, in theca cells, it promotes androgen production [72]. During the intermediate follicle phase, LH contributes to follicular recruitment thereby increasing FSH-R expression in granulosa cells. Together with FSH, LH induces local growth factors involved in follicular maturation [73–75]. Lastly, during ovulation, LH exerts several essential functions involved in oocyte quality, for instance, it induces the completion of meiosis and extrusion of the first polar body [76].

In most women undergoing OS for ART, endogenous LH sustains follicle recruitment thereby obviating the need for exogenous supplementation during this process. However, advanced age women have a “relative” deficiency of the LH system. Indeed, despite a normal LH level [77], these women exhibit decreased LH activity, which in turn impairs androgen production. Indeed, levels of circulating androgens were found to be much lower in older than in younger reproductive age women. Zumoff et al. reported that the expected testosterone level of a 40 year old woman is about half that of a 21 year old woman [8]. In line with this finding, Mushayandebvu et al. observed reduced androstenedione and testosterone levels at midcycle in older reproductive age women [78]. A steep decrease in circulating androgens in late reproductive age was confirmed by a large cross sectional study involving 1423 women in whom total testosterone, free testosterone, DHEA-S and androstenedione declined dramatically after the age of 25 years [7]. It has been suggested that the androgen serum deficiency observed in advanced age could be compensated by androgen supplementation [79, 80]. However, it seems implausible that androgen supplementation could affect the intrafollicular androgen milieu given that serum androgen concentrations do not correlate with levels of androgen follicle fluid even in advanced age women [81, 82]. In contrast, LH supplementation induces local follicle androgen production and might be a more physiological approach with which to modulate follicular function than androgen supplementation [29, 81].

While it seems conceivable that LH supplementation could mitigate the age-related impairment of both androgen production and androgen follicular fluid levels [30], the age-related aneuploidy rate, which becomes increasingly more relevant in women above the age of 40 years [34], seems to be unavoidable regardless of the type or dose of the gonadotropin regimen used. This hypothesis partially explains why the impact of r-hFSH/r-hLH co-treatment disappeared when women above 40 years old were included in the analysis.

The impaired function of the LH system seen in ageing women could also be exacerbated by the pituitary suppression regimen used during OS. Indeed, both GnRH agonist and GnRH antagonist regimens dramatically decrease the circulating LH level in peripheral blood of women undergoing IVF, regardless of age [48, 57, 83]. In line with this finding, Younis et al. [84] reported a relative LH deficiency in older women undergoing a flexible antagonist regimen. They also reported that r-hFSH/r-hLH co-treatment could counteract this deficiency.

In addition, a pilot study demonstrated that r-hFSH/r-hLH co-treatment significantly improved implantation rates and the number of positive pregnancy tests in women with a history of repeated implantation failure [85]. This effect seems mainly due to an increased oocyte quality and the anti-apoptotic effect exerted by LH on cumulus cells [62]. In addition, LH acts through post-receptor paracrine signaling thereby promoting cell expansion and oocyte maturation during folliculogenesis [86]. In our meta-analysis, we observed that, although fewer oocytes were retrieved in the r-hLH-r-hFSH co-treatment group than in the r-hFSH-only group, the number of MII oocytes in the co-treatment group was comparable to that in the r-hFSH-only group, and the clinical pregnancy rate was significantly better in the co-treatment group. These findings suggest that the addition of r-hLH to hFSH in women at an advanced reproductive age might improve the quality rather than the quantity of ovarian response. A positive effect of r-hLH addition on live birth rate and miscarriage rate has yet to be established. This is not surprising given that data about live births were reported in only 22% of trials of ART treatment [87]. Similarly, data about pregnancy loss was not reported in 33% of infertility treatment trials [87]. These deficiencies are mainly related to the fact that pregnancies are usually cared for by physicians other than those who performed the IVF process. In addition, follow-up of pregnancies considering all possible confounders (e.g., gestational complications unrelated to IVF) until delivery (approximately 40 weeks after transfer) could be challenging and costly for both sponsored and unsponsored clinical trials [87]. The lower number of oocytes retrieved in the r-hLH co-treated group is consistent with a previous meta-analysis [88] and supports the evidence that LH could suppress small preovulatory follicles thereby favoring maturation of the largest ones during OS [89].

Lastly, LH endometrial maturation may be disturbed in case of LH deficiency [90]. Indeed, LH modulates several signaling molecules involved in endometrial implantation, such as cytokine leukemia inhibiting factor, colony-stimulating factor-1 and interleukin-1, integrins, glycodelin and mucin 1 [90, 91]. Furthermore, in vivo studies demonstrated that the LH receptor is increased at the time of implantation, which suggests that LH signaling may contribute to implantation [92].

### Limitations

The main limitation of this review and meta-analysis is that very few studies have analyzed the clinical effect of r-hFSH/r-hLH co-treatment within a specific age range. As mentioned in the Introduction, the IVF prognosis in terms of live birth dramatically changes year by year after 35 years of age, irrespective of the treatment strategy used [1]. This explains the inconsistent findings observed when studies that enrolled patients with a large age range (for instance ≥35 years) [36, 37] were compared with studies using a narrow age range (for instance 35–39 years) [9, 64]. We believe that large studies with a narrow age range would better elucidate the role of r-hLH in women of advanced reproductive age.

Another issue is the fact that only two studies reported live birth data, which is considered the most relevant endpoint in reproductive medicine. However, data about clinical pregnancy rates, which has been addressed in a large number of trials, suggest that women between 35 and 40 years of age could benefit from r-hLH supplementation. Similar to live birth rate, clinical pregnancy rate could be a reliable parameter of IVF success [93, 94]. Indeed, in a meta-analysis of 143 RCTs, Clarke and colleagues demonstrated that conclusions regarding the effectiveness of a treatment based on either clinical pregnancy or live birth as endpoints are comparable (kappa 0.81; 95% CI: 0.68–0.94) [94].

Notably, live birth as the primary endpoint could be debatable in advanced age patients given the age-dependent miscarriage rate observed in these patients [33]. For example, the miscarriage rate in women over 40 years was estimated to be approximately 30% [33]. Not surprisingly, a dramatic pre-delivery drop-out has been observed during similar trials, and the sample size required to analyze this endpoint is usually economically unsustainable [93]. This explains why, even the largest RTCs published so far were not sufficiently powered to detect differences in terms of live birth rates between treatment arms [37, 71]. In most cases the power analysis was made on clinical pregnancy rate and the number of oocytes, respectively [37, 67, 71]. The process of converting a secondary outcome such as live birth rate from original trials to a primary outcome in meta-analyses inevitably leads to a Type I error [95]. Furthermore, live birth rate is more prone to bias than the clinical pregnancy rate and is unrelated to the type of gonadotropins used for OS in ART. For instance, intrauterine fetal death after 12 weeks of gestation occurs in about 5% of ongoing pregnancies after IVF, and this risk increases in women of advanced age. This phenomenon is not related to the type of gonadotropin treatment administered but depends essentially on the increased aneuploidy rate of the embryos recorded in advanced age women [34, 96]. Furthermore, the association between advanced maternal age per se and fetal death is well established and is independent of maternal morbidity and ART treatment [97]. Given the foregoing, we believe that clinical pregnancy rate is a more relevant and reliable main outcome measure than live birth rate.

Lastly, there is still some controversy regarding the dosage and timing of r-hLH administration. Several regimens have been proposed. Some authors suggested starting LH upon the onset of OS treatment [9, 63], others starting from the intermediate phase of OS [36, 37]. In the first phase of folliculogenesis, LH receptors are expressed predominantly in theca cells thereby promoting ovarian androgen production, which, as mentioned above, is reduced in advanced age women [7]. The latter finding might explain why implantation and clinical pregnancy rates were better in advanced age women when r-hLH was prescribed with r-hFSH from the beginning of OS than when r-hLH was added to r-hFSH therapy during OS [9, 63]. On the other hand, it is widely acknowledged that LH receptors are expressed in granulosa cells starting from the intermediate follicular phase, and that LH can support follicular growth [98, 99]. Consistently, several data demonstrate that LH could resolve the follicular growth stagnation that characterizes young women with a hypo-response. Indeed, in these patients, the administration of r-hLH during OS (i.e., stimulation day 7 or 8) significantly increased the number of oocytes retrieved [100]. Regarding dosage, a 2:1 ratio with r-hLH between 75 IU and 150 IU administered from the onset of stimulation appears to be sufficient to obtain a clinical benefit in women between the ages of 35–39 years [9, 63].

### Future research

As mentioned above, the conflicting results reported so far regarding the effect of r-FSH/r-hLH co-treatment in advanced age women are pivotally linked to the wide age range used in most trials involving advanced age women [36, 37]. Herein we have partially addressed this issue by performing a subgroup analysis of studies with a narrower age-range, namely those involving women between 35 and 40 years of age. In the attempt to strengthen our findings, we adopted a more conservative approach, using the random-effect model for all outcomes analyzed. However, we recognize that further research is needed in women between the ages of 35–40 years, and of women in narrower age ranges (i.e., 35–37 and 38–40 years) to understand better the benefit of r-hFSH/r-hLH co-treatment. Future studies should consider administering r-hFSH/r-hLH co-treatment from the start of OS because this regimen appears to be better than when it is started 6–7 days after OS with FSH alone [9, 63].

## Conclusion

This meta-analysis shows that r-hFSH/r-hLH co-treatment and r-hFSH monotherapy have comparable clinical pregnancy rates in women above the age of 34 years. However, women between 35 and 40 year old, might benefit from r-hFSH/r-hLH co-treatment in terms of clinical pregnancy and implantation rates. Further research using narrower age-ranges is warranted to corroborate these findings.

## Supplementary Information

**Additional file 1: Supplemental Table 1.** Sensitivity analysis.

**Additional file 2: Supplemental Figure 1.** Risk of bias per study and summary.

**Additional file 3: Supplemental Figure 2.** Forest plot showing the effect of r-hFSH + r-hLH versus r-hFSH monotherapy in ovarian stimulation on live birth rate.

**Additional file 4: Supplemental Figure 3.** Forest plot showing the effect of r-hFSH + r-hLH versus r-hFSH monotherapy in ovarian stimulation on the miscarriage rates: a) age ≥ 35 years b) between 35 and 40 years old.

**Additional file 5: Supplemental Figure 4.** Funnel-plots and “trim and fill” analysis of primary outcome. White dots represent the values observed in each study while black dots represent studies “trimmed” for funnel plot asymmetry. The white diamond represents the overall observed effect size (OR 1.11, 95% CI 0.89–1.38) while the black diamond represents the overall effect size using trim and fill method (OR 1.05, 95% CI 0.85–1.29). The trim and fill analysis showed there was no substantial changes in the effect size of the primary outcome.

## Data Availability

Not applicable.
