# Supplementary material for: Recombinant human luteinizing hormone co-treatment in ovarian stimulation for assisted reproductive technology in women of advanced reproductive age: a systematic review and meta-analysis of randomized controlled trials
Source: Reprod Biol Endocrinol. 2021 Jun 21;19:91. doi: 10.1186/s12958-021-00759-4 (PMC8215738; doi:10.1186/s12958-021-00759-4)
Supplement: Supplementary file 1 — Additional file 1: Supplemental Table 1. Sensitivity analysis. [file 12958_2021_759_MOESM1_ESM.docx]

| **Outcome** | **All studies** |  | | **Sensitivity analysis^a^** | | | **Results** |
| --- | --- | --- | --- | --- | --- | --- | --- |
|  | **N. Studies/**  **participants** | **Effect size [95%CI]** | **I^2^** | **N. Studies/**  **participants** | **Effect size [95%CI]** | **I^2^** |  |
| Clinical pregnancy rate | 11/1670 | 1.11 [0.89-1.38] | 2% | 7/1234 | 1.25 [0.97-1.60] | 0% | Not affected |
| N. oocytes retrieved | 11/2266 | -0.87 [-1.47; -0.28] | 74% | 7/1896 | -0.91 [-1.67; -0.15] | 83% | Not affected |
| N. Metaphase II oocytes | 7/934 | -0.35 [-1.10; -0.40] | 84% | 4/664 | -0.32 [-1.52; 0.88] | 90% | Not affected |
| Implantation rate | 10/1605 | 1.16 [0.92-1.46] | 24% | 6/1113 | 1.29 [0.99-1.68] | 23% | Not affected |
| Live birth rate | 2/371 | 1.53 [0.50-4.65] | 67% | 2/371 | 1.53 [0.50-4.65] | 65% | Not affected |
| Miscarriage rate | 7/958 | 1.12 [0.64;1.95] | 0% | 4/652 | 1.01 [0.51-0.99] | 0% | Not affected |
| ^a^ Barrentexea et al. 2008 excluded for unclear Attrition bias and Reporting bias;  Fabregues et al. 2011 excluded for high Attrition bias  Nyboe Andersen et al. 2008 excluded for unclear Selection bias and high Reporting bias  Younis et al. 2016 excluded for unclear risk of Selection bias and high risk of Performance bias | | | | | | | |
