# Supplementary figures and images for: Recombinant human luteinizing hormone co-treatment in ovarian stimulation for assisted reproductive technology in women of advanced reproductive age: a systematic review and meta-analysis of randomized controlled trials
Source: Reprod Biol Endocrinol. 2021 Jun 21;19:91. doi: 10.1186/s12958-021-00759-4 (PMC8215738; doi:10.1186/s12958-021-00759-4)

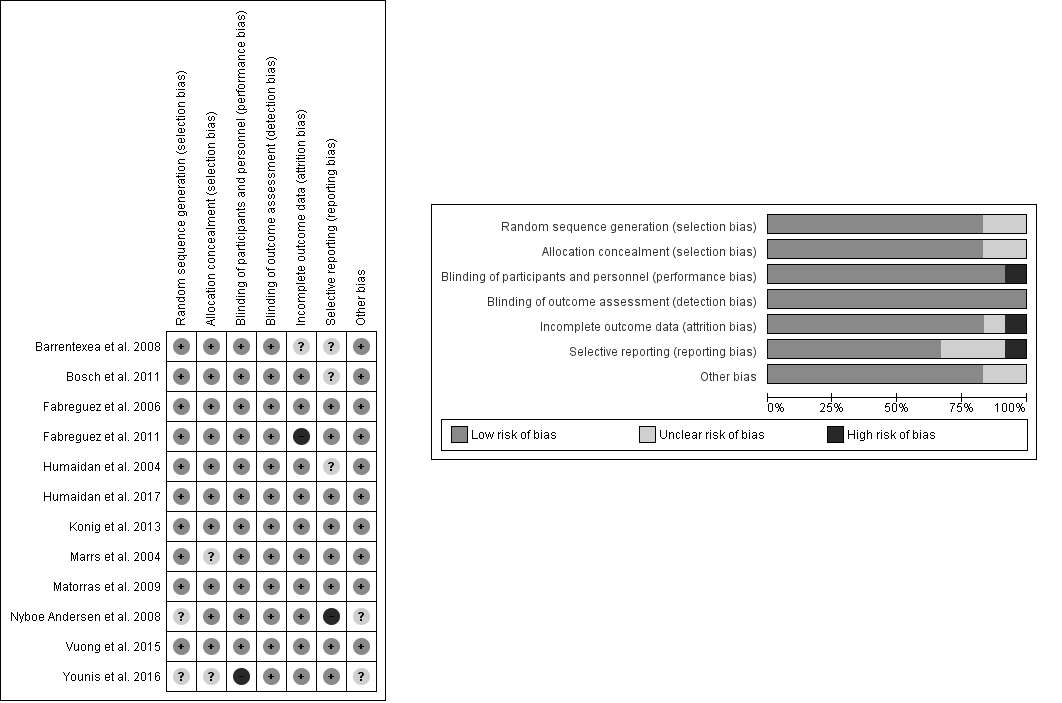

Supplement: Supplementary file 2 — Additional file 2: Supplemental Figure 1. Risk of bias per study and summary. [file 12958_2021_759_MOESM2_ESM.jpg]

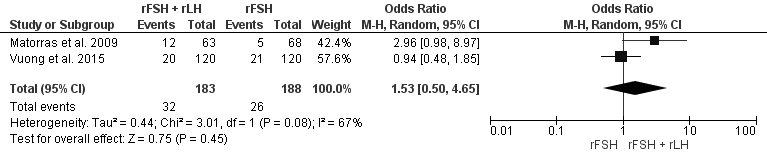

Supplement: Supplementary file 3 — Additional file 3: Supplemental Figure 2. Forest plot showing the effect of r-hFSH + r-hLH versus r-hFSH monotherapy in ovarian stimulation on live birth rate. [file 12958_2021_759_MOESM3_ESM.jpg]

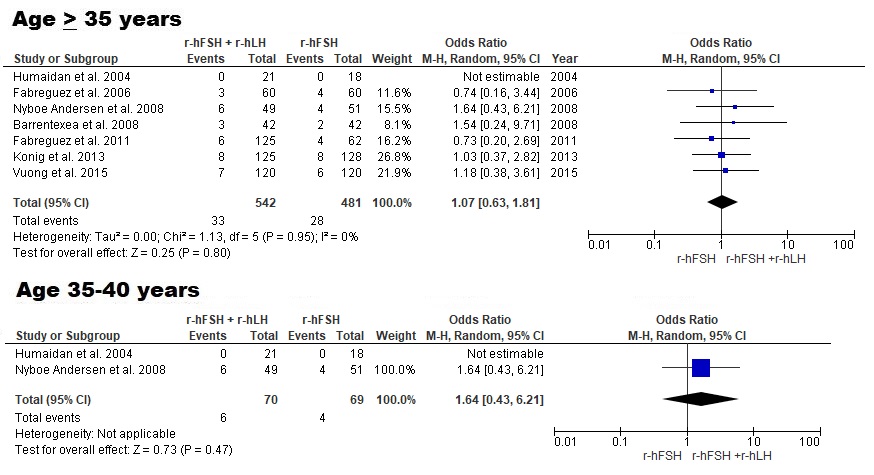

Supplement: Supplementary file 4 — Additional file 4: Supplemental Figure 3. Forest plot showing the effect of r-hFSH + r-hLH versus r-hFSH monotherapy in ovarian stimulation on the miscarriage rates: a) age ≥ 35 years b) between 35 and 40 years old. [file 12958_2021_759_MOESM4_ESM.jpg]

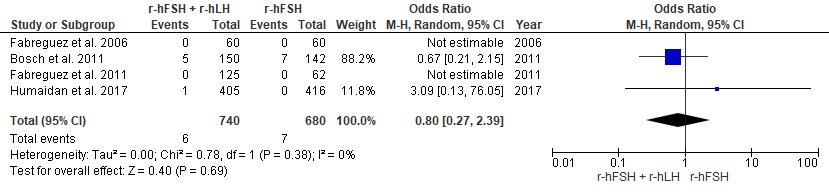

Supplement: Supplementary file 5 — Additional file 5: Supplemental Figure 4. Funnel-plots and “trim and fill” analysis of primary outcome. White dots represent the values observed in each study while black dots represent studies “trimmed” for funnel plot asymmetry. The white diamond represents the overall observed effect size (OR 1.11, 95% CI 0.89–1.38) while the black diamond represents the overall effect size using trim and fill method (OR 1.05, 95% CI 0.85–1.29). The trim and fill analysis showed there was no substantial changes in the effect size of the primary outcome. [file 12958_2021_759_MOESM5_ESM.jpg]
